# Model and Task-Aware Test-Time Scaling Strategies for Large Language and Vision-Language Models in Medicine: Evaluation Study

**DOI:** 10.2196/90693

**Published:** 2026-07-23

**Authors:** Gyutaek Oh, Seoyeon Kim, Sangjoon Park, Byung-Hoon Kim

**Affiliations:** 1 Department of Biomedical Systems Informatics, College of Medicine, Yonsei University, 50-1 Yonsei-ro, Seodaemun-gu, Seoul, 03722, Republic of Korea, 82 2-2228-2484; 2 Yonsei Institute for Digital Health, Seoul, Republic of Korea; 3 College of Medicine, Yonsei University, Seoul, Republic of Korea; 4 Department of Radiation Oncology, College of Medicine, Yonsei University, Seoul, Republic of Korea; 5 Heavy Ion Therapy Research Institute, Yonsei University College of Medicine, Seoul, Republic of Korea; 6 Yonsei Cancer Center, Yonsei University College of Medicine, Seoul, Republic of Korea; 7 Department of Psychiatry, College of Medicine, Yonsei University, Seoul, Republic of Korea; 8 Institute of Behavioral Sciences in Medicine, Yonsei University College of Medicine, Seoul, Republic of Korea

**Keywords:** test-time scaling, large language models, vision-language models, reasoning models, medical AI, clincal reasoning

## Abstract

**Background:**

Test-time scaling has emerged as a promising method to enhance the reasoning capabilities of large language models (LLMs) and vision-language models (VLMs) during inference without additional training. While foundational studies established scaling paradigms in general domains, their applicability to the unique complexities of medical AI remains underexplored.

**Objective:**

This study aims to conduct a comprehensive investigation of test-time scaling in the medical domain. We evaluate the impact of scaling across different model sizes and task complexities. Furthermore, we seek to identify domain-specific bottlenecks and assess model robustness against user-driven perturbations, such as misleading clinical authority.

**Methods:**

This study evaluated a diverse set of general and medical-specific LLMs and VLMs. Experiments used five textual medical benchmarks comprising over 5500 questions and two multimodal benchmarks comprising 7000 samples. Performance was measured under three scaling conditions: increasing token budgets, iterative sequential scaling, and parallel scaling. Robustness was tested by embedding misleading hints with varying tones and levels of simulated clinical expertise into prompts.

**Results:**

For nonreasoning LLMs, accuracy saturated quickly, with token usage often remaining under 500 tokens regardless of budget increases. Reasoning models demonstrated significant performance gains on complex tasks as token budgets increased. Notably, we identified distinct domain-specific behaviors. First, current VLMs showed a structural bottleneck in integrating visual clues and experienced limited benefit from token expansion. Second, medically fine-tuned LLMs excelled in clinical question answering but exhibited degraded scaling efficiency on calculation tasks compared to general-domain models. This reflects a disparity between qualitative clinical alignment and procedural logic. Third, while optimal scaling improved robustness, models exhibited a cognitive vulnerability by readily abandoning correct reasoning when confronted with misleading expert physician hints. Regarding scaling strategies, parallel scaling outperformed sequential scaling on easier tasks. Conversely, extended sequential scaling or increased budgets proved essential for complex problem-solving.

**Conclusions:**

Test-time scaling rules from general domains do not perfectly translate to medical AI. Longer reasoning is not universally beneficial. Concise reasoning with parallel scaling is optimal for simpler tasks. An extended chain of thought via sequential scaling or increased budgets is required for complex problems. Furthermore, safe clinical deployment requires addressing fundamental vision-language alignment, balancing clinical and procedural reasoning, and mitigating vulnerabilities to perceived clinical authority.

## Introduction

### Background

In recent years, large language models (LLMs) have undergone rapid development.

Since the introduction of OpenAI’s GPT-3 [1], both industry and academia have actively competed to develop powerful LLMs [2-9], which are now widely adopted in daily life. More recently, models that integrate data from multiple modalities, particularly vision-language models (VLMs) [10-16], have garnered significant attention among multimodal approaches.

A common trend in developing these models has been to scale up both model size and training data in pursuit of improved performance. However, the high demand for computational resources and massive datasets presents substantial barriers to broader accessibility and development. Moreover, while training-time scaling laws have led to certain improvements, many models still struggle with complex reasoning tasks.

To address these challenges, test-time scaling has recently emerged as an effective method for enhancing LLM performance during inference [17-20]. For instance, rather than generating an immediate final prediction, a model can be configured to produce intermediate step-by-step logical deductions or generate multiple candidate responses to find a reliable consensus. Without requiring additional training or fine-tuning, test-time scaling improves both reliability and accuracy, especially on tasks that require multistep reasoning. By increasing the token budget or generating multiple candidate responses, test-time scaling enables models to produce more detailed and structured chain-of-thought reasoning. This approach is particularly synergistic with reasoning-optimized models, which are trained using supervised fine-tuning (SFT) on chain-of-thought–annotated datasets or reinforcement learning (RL) with specialized objectives [21-23]. The combination of reasoning models and test-time scaling has shown state-of-the-art results on complex tasks such as mathematics problem solving and program code synthesis [6,9].

Recently, applications of LLMs and VLMs in the medical domain have also gained momentum [24-30]. Alongside these applications, both reasoning models [29-33] and test-time scaling [30,34,35] are being increasingly adopted in medical AI research. Recent studies demonstrate that the use of reasoning models and test-time scaling significantly boosts performance on medical benchmark datasets [30].

Despite growing interest in test-time scaling in the medical domain, many important aspects remain underexplored. Although various test-time scaling strategies have been proposed, only a limited subset has been systematically evaluated in medical applications. For example, it remains unclear whether shorter or longer reasoning is more advantageous in the medical domain, or whether sequential or parallel scaling is more effective. Furthermore, despite differences in training data and methods across models, many prior studies have applied test-time scaling strategies uniformly, without accounting for each model’s unique characteristics. Given the wide range of difficulty in medical tasks, it is also important to examine whether reasoning via test-time scaling is equally beneficial across different task types. In addition, with the increasing relevance of VLMs in clinical and diagnostic contexts, investigating test-time scaling strategies for VLMs in the medical domain is essential.

Crucially, while foundational studies have established test-time scaling paradigms in the general domain by focusing primarily on text-based logic such as mathematics and coding [18-20], their findings cannot readily predict the multilayered complexities inherent to clinical AI. General-domain frameworks fail to capture the domain-specific bottlenecks and unique behavioral shifts that emerge in medical environments. Specifically, it remains unexamined how test-time compute interacts with the intricate visual reasoning required for complex medical images, whether specialized medical knowledge fine-tuning paradoxically compromises a model’s baseline numerical reasoning in clinical calculations, and how scaling affects a model’s cognitive vulnerability when confronted with incorrect information embedded in user prompts. Resolving these questions is vital to discovering insights unique to medicine that transcend mere empirical replication of general-domain findings.

**Figure 1.**
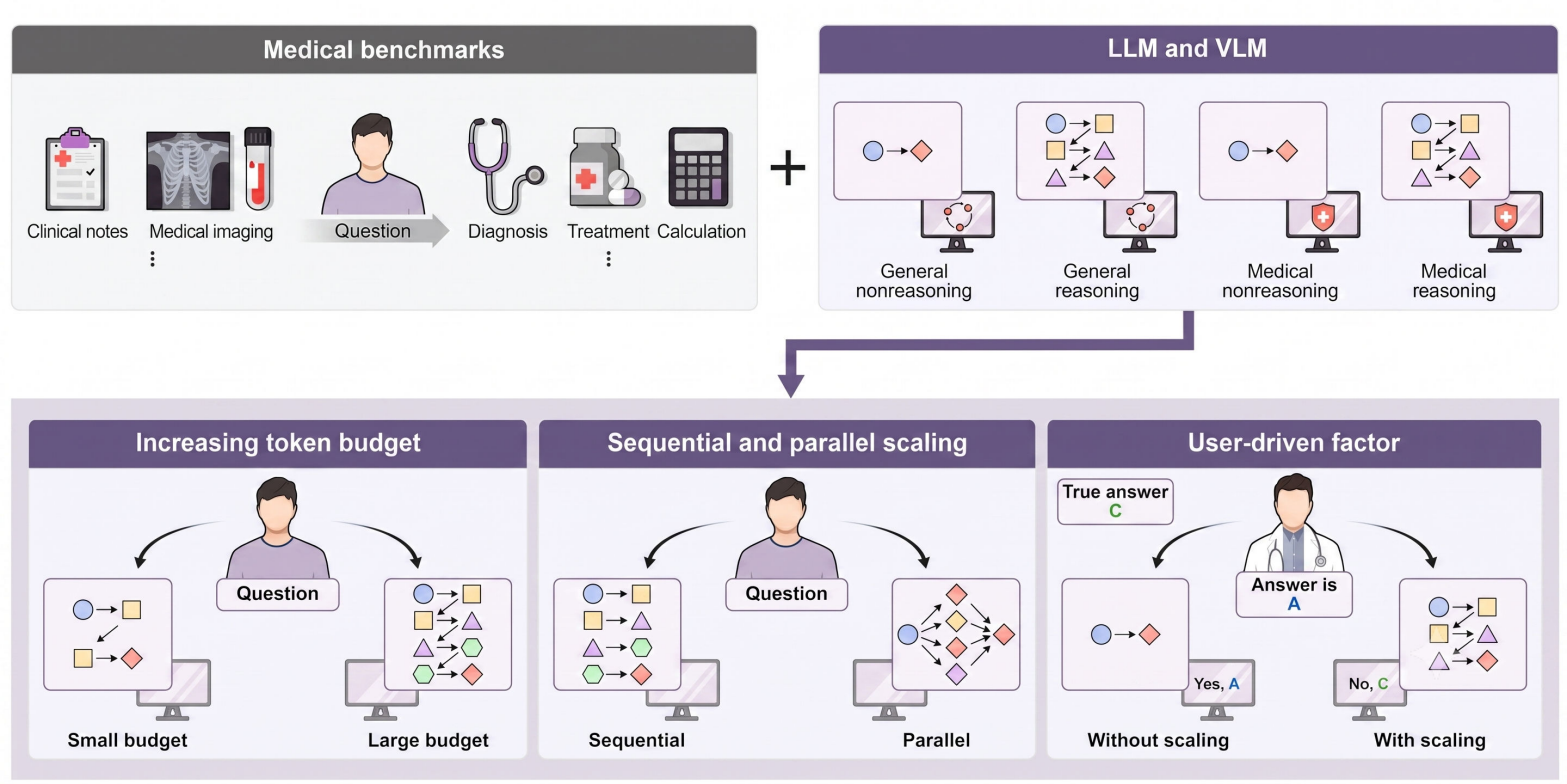
Overview of our study: investigating test-time scaling for LLMs and VLMs in medicine. LLM: large language model; VLM: vision-language model.

To bridge these gaps, in this paper, we present a comprehensive investigation of test-time scaling for medical applications. Figure 1 illustrates the overall framework of our study. First, we analyze how increasing the token budget affects the performance of various LLMs and VLMs across multiple medical benchmark datasets. We explore how this performance varies with factors such as model size, model characteristics, and task complexity. Next, we compare sequential and parallel test-time scaling strategies, highlighting their relative effectiveness in medical tasks. Finally, we evaluate the robustness of test-time scaling under user-driven factors, such as misleading contextual information embedded in prompts. Ultimately, the primary aim of this study is to establish a domain-specific framework for scaling inference compute in medical AI. We hypothesize that the optimal scaling strategy is fundamentally dependent on both the inherent reasoning capacity of the model and the specific complexity of the clinical task, and that appropriate scaling can significantly improve model robustness against misleading user inputs.

### Prior Work

#### Test-Time Scaling

Test-time scaling has recently emerged as a method for enhancing the reasoning capabilities of LLMs during inference by increasing the number of tokens used for chain-of-thought reasoning. Following the introduction of OpenAI’s o1 model [7], which demonstrated strong performance on complex tasks through enhanced chain-of-thought prompting, many subsequent studies have begun to explore test-time scaling as a means of improving LLM performance without additional training.

The s1 model [17] introduces test-time scaling through “budget forcing,” controlling computational effort during inference by appending “wait” tokens to extend thinking or forcefully terminating the response. Remarkably, s1 uses only 1000 curated questions with reasoning paths selected for difficulty, diversity, and quality, yet exceeds the o1-preview model on competition mathematics questions by up to 27%. On the other hand, subsequent studies [20] revealed that longer chain-of-thought responses do not consistently enhance accuracy, where correct solutions are often shorter than incorrect ones. This phenomenon suggests that excessive self-revision may degrade model performance rather than enhance it.

In response to these findings, recent studies have proposed test-time scaling strategies that either extend chain-of-thought reasoning sequentially or generate multiple candidate responses in parallel, aiming to discover optimal configurations that balance depth and diversity of reasoning. In the study by Balachandran et al [36], it was demonstrated that both sequential and parallel scaling can improve the performance of nonreasoning and reasoning models. For instance, parallel scaling significantly boosted accuracy on the Traveling Salesman Problem easy subset, improving performance from 42% to 95% when scaled to 256 parallel API calls. More broadly, Snell et al [18] emphasized that identifying the optimal test-time scaling strategy according to task difficulty is crucial, as it can yield greater performance gains than increasing model parameters. Beyond language models, recent research has demonstrated that efficient test-time scaling can also significantly enhance the capabilities of small VLMs [37].

#### LLMs and VLMs for the Medical Domain

The medical domain has advanced through specialized models using various training paradigms. UltraMedical [27] provides a suite of biomedical LLMs fine-tuned on 410,000 high-quality instructions with preference annotations, achieving state-of-the-art performance through SFT and iterative preference learning. HuatuoGPT-o1 [29] uses medical problems with a medical verifier to guide complex reasoning and RL, outperforming baselines using only 40K problems.

Extending test-time scaling to medicine, m1 [34] adapts s1’s methodology using small datasets with reasoning traces and thinking token budgets, enabling lightweight models under 10B parameters to achieve state-of-the-art medical reasoning with a 4K token budget.

To achieve comprehensive medical understanding, multimodal models have recently emerged. HuatuoGPT-Vision [38] integrates visual and textual medical knowledge as a 34B multimodal LLM trained on 1.3 million medical VQA (visual question answering) samples. MedGemma [39] is Google’s open-source medical AI collection combining multimodal capabilities, available in 4B multimodal built on Gemma 3 architecture.

Beyond architecture, RL has been increasingly leveraged for test-time scaling and improved model robustness in vision-language medical models. MedVLM-R1 [33] uses RL to generate natural language reasoning alongside answers. Med-R1 [32] uses group relative policy optimization [23] to improve generalizability across various medical imaging modalities, achieving a 29.94% accuracy improvement. Both models demonstrate RL effectiveness in medical AI, with MedVLM-R1 focusing on reasoning transparency and Med-R1 emphasizing cross-modality generalization.

## Methods

### Datasets

In our experiments, we evaluate LLMs on five medical benchmark datasets, including four medical question answering (QA) datasets and one medical calculation dataset. Characteristics of all medical benchmark datasets used in this study are summarized in Table 1.

**Table 1.** Medical benchmark datasets for our experiments.

Name	Type	Description	Answer format	Samples (n)	Difficulty
Text-only					
PubMedQA	Medical QA^a^	Research questions with corresponding abstracts	One of yes/no/maybe	500	Easy
MedQA	Medical QA	Questions based on the USMLE^b^	One of five options (A to E)	1273	Intermediate
MedBullets	Medical QA	Questions based on the USMLE	One of five options (A to E)	308	Intermediate
MedXpertQA (text)	Medical QA	Expert-level examination questions	One of ten options (A to J)	2450	Difficult
MedCalc-Bench	Medical calculation	Patient notes and corresponding questions	Decimal, integer, date, time	1047	Difficult
Vision-text					
OmniMedVQA	Medical VQA^c^	Images from various modalities and corresponding questions	One of two/three/four options	5000	Easy
MedXpertQA (multimodal)	Medical VQA	Expert-level examination questions with corresponding images	One of five options (A to E)	2000	Difficult

a QA: question answering.

b USMLE: United States Medical Licensing Examination.

c VQA: visual question answering.

PubMedQA [40] is a medical QA dataset comprising 500 research questions, each paired with a relevant abstract. The model must answer each question with one of three choices: “yes,” “no,” or “maybe.”

MedQA [41] includes medical QA pairs derived from textbooks. In our study, we use a subset of 1273 multiple-choice questions based on the USMLE (United States Medical Licensing Examination), each with five answer options.

MedBullets [42] is another USMLE-style medical QA dataset, consisting of 308 multiple-choice questions, each with five options, similar in format to MedQA.

MedXpertQA (text) [43] is a recently proposed medical QA benchmark that includes both textual and multimodal (text and image) questions. For our LLM experiments, we use only the text-based subset, which contains 2450 questions with ten answer choices each.

MedCalc-Bench [44] is a medical calculation benchmark comprising 1047 questions, each accompanied by a corresponding patient note. The dataset is designed to evaluate models’ ability to perform clinical calculations based on contextual patient information.

We define the difficulty of the medical QA datasets in ascending order as follows: PubMedQA, MedQA, MedBullets, and MedXpertQA, based on previous studies [34,35,40-45], the number of choices, and the level of reasoning required to answer the questions. Additionally, given that prior studies have demonstrated the substantial reasoning demands involved in calculations [17-20], we consider MedCalc-Bench a difficult dataset that requires extensive reasoning.

Next, we evaluate VLMs on two medical multimodal datasets.

OmniMedVQA [46] is a large-scale benchmark designed for evaluating medical VLMs. OmniMedQVA consists of simple and short questions based on provided images, making its overall difficulty relatively easy. For our experiments, we randomly sample 5000 questions spanning three categories that require relatively more reasoning: disease diagnosis, lesion grading, and other biological attributes.

Regarding MedXpertQA (multimodal), for our VLM experiments, we use a multimodal subset of the MedXpertQA dataset, which includes 2000 multiple-choice questions, each with five answer options. Each question is accompanied by one to six associated medical images. As the models have to process multiple images simultaneously and comprehend the contextual information in the questions, we consider MedXpertQA a challenging task.

### Models

We use the following LLMs for our experiments.

Regarding general instruction-tuned LLMs, we evaluate instruction-tuned versions of Llama 3 [5] (Llama 3-3B, 8B, 70B) and Qwen2.5 [8] (Qwen2.5-3B, 7B, 32B, 72B) as representative general-purpose LLMs. Both models have been fine-tuned on instruction-following datasets. Llama 3 is trained using SFT followed by RL from human feedback [47], whereas Qwen2.5 is fine-tuned using a combination of SFT, direct preference optimization [22], and group relative policy optimization [23]. In addition, we evaluate the Qwen3-Instruct models (Qwen3-8B, 30B-IT), which represent the updated nonthinking mode of the Qwen3 model [48].

Regarding general reasoning LLMs, we also evaluate the Qwen3-Thinking models (Qwen3-8B, 30B-TH), which are scaled to enhance the thinking capability of the Qwen3 models, specifically targeting improved quality and depth of reasoning [48]. Next, we include distilled versions of DeepSeek-R1 [9] (DeepSeek-R1-7B, 8B, 32B, 70B) as representative models optimized for general reasoning capabilities. While the original DeepSeek-R1 is trained using both SFT and RL, the distilled versions are trained solely via SFT using a reasoning dataset generated by the original DeepSeek-R1 model. Finally, we include the gpt-oss models [49] (gpt-oss-20B, 120B), OpenAI’s open-source series trained via SFT and RL.

Regarding medical LLMs, we evaluate LLMs specifically trained for the medical domain.

UltraMedical [27] is a medical LLM fine-tuned using SFT followed by preference optimization techniques such as direct preference optimization [22] or Kahneman-Tversky optimization [50]. We use the 8B and 70B variants of UltraMedical in our experiments (UltraMedical-8B, 70B).

Regarding medical reasoning LLMs, we also evaluate LLMs specifically designed for medical reasoning tasks. HuatuoGPT-o1 [29] (HuatuoGPT-7B, 8B, 70B, 72B) is a medical LLM trained on synthetic medical problems featuring complex chain-of-thought reasoning. The model is trained using SFT and RL via proximal policy optimization [21].

In addition, we include m1 [34], a medical reasoning model trained with curated chain-of-thought–style data using SFT, but without RL. In our experiments, we use versions of m1 that are trained on a dataset of 1000 samples, which are denoted as m1-7B, 32B in this paper.

Regarding general VLMs, we evaluate five general-purpose VLMs: Llama 3-Vision [5] (Llama 3-Vision-11B, 90B), Qwen2.5-VL [51] (Qwen2.5-VL-7B, 32B), Qwen3-VL [52] (Qwen3-VL-8B, 30B-IT), Gemma 3 [53] (Gemma 3-12B, 27B), and LLaVA [13] (Large Language and Vision Assistant; LLaVA-7B, 13B). For Llama, Qwen, and Gemma models, we use instruction-tuned versions.

Regarding general reasoning VLMs, we also include general-domain models explicitly trained for reasoning tasks. First, the thinking version of Qwen3-VL [52] (Qwen3-VL-8B, 30B-TH) is evaluated. Next, LLaVA-CoT [54] (Large Language and Vision Assistant–Chain-of-Thought; LLaVA-CoT-11B) is a VLM fine-tuned with synthetic chain-of-thought data using SFT. Finally, we evaluate the preview version of QVQ [55] (Qwen With Vision and Questions; QVQ-72B), a large-scale multimodal reasoning model built upon Qwen2-VL-72B [15].

Regarding medical VLMs, we evaluate VLMs specifically tuned for the medical domain. MedGemma [39] (MedGemma-4B, 27B) is a medical variant of Gemma 3 trained on medical text and images. In our experiment, we use the instruction-tuned version of MedGemma. HuatuoGPT-Vision [38] (HuatuoGPT-Vision-7B, 34B) is another medical VLM fine-tuned on medical VQA data.

Regarding medical reasoning VLMs, lastly, we include the medical reasoning VLM, QoQ-Med [31], in our evaluation. QoQ-Med is trained on the CLIMB (clinical large-scale integrative multimodal benchmark) [56], a comprehensive dataset that incorporates diverse types of medical data, including ECG (electrocardiogram; 1D), chest X-rays (2D), and magnetic resonance imaging scans (3D). To enhance multimodal reasoning capabilities, the authors of a study [31] proposed a domain-aware group relative policy optimization, which applies hierarchical scaling strategies based on the domain of the input data. In our experiments, we evaluate two versions of QoQ-Med: QoQ-Med-7B and QoQ-Med-32B.

Table 2 summarizes the models used in our experiments and their key characteristics. In our experiments, we use a 4-bit quantized version of the models when the number of model parameters is 70B or more.

**Table 2.** LLMs^a^ and VLMs^b^ for our experiments.

Model name	Model type	Domain	Base model	Dataset	Training method	Model size
LLM						
Llama 3-Instruct	General	Nonreasoning	Llama 3	Instruction datasets	SFT^c^+RLHF^d^	3B, 8B, 70B
Qwen2.5-Instruct	General	Nonreasoning	Qwen2.5	Instruction datasets	SFT+DPO^e^+GRPO^f^	3B, 7B, 32B, 72B
Qwen3-Instruct	General	Nonreasoning	Qwen3	Unknown	Unknown	4B, 30B (3B activated)
Qwen3-Thinking	General	Reasoning	Qwen3	Unknown	Unknown	4B, 30B (3B activated)
DeepSeek-R1-Distill	General	Reasoning	Llama 3 or Qwen2.5	Reasoning data generated by DeepSeek-R1	SFT	7B, 8B, 32B, 70B
gpt-oss	General	Reasoning	N/A^g^	Text-only dataset with a focus on STEM^h^, coding, and general knowledge	SFT+CoT^i^ RL^j^	20B, 120B
UltraMedical	Medical	Nonreasoning	Llama 3	Synthetic (by GPT-4 [2]) and manually curated medical data	SFT+DPO or KTO^k^	8B, 70B
HuatuoGPT-o1	Medical	Reasoning	Llama 3 or Qwen2.5	Medical data with synthetic CoT reasoning (by GPT-4o [6])	SFT+PPO^l^	7B, 8B, 70B, 72B
m1	Medical	Reasoning	Qwen2.5	Curated medical data with synthetic reasoning (by DeepSeek-R1)	SFT	7B, 32B
VLM						
Llama 3-Vision-Instruct	General	Nonreasoning	Llama 3-Vision	Vision-language instruction dataset	SFT+RLHF	11B, 90B
Qwen2.5-VL-Instruct	General	Nonreasoning	Qwen2.5-VL	Vision-language instruction dataset	SFT+DPO	7B, 32B
Gemma 3-it	General	Nonreasoning	Gemma 3	Vision-language instruction dataset	SFT+RLHF	12B, 27B
LLaVA^m^	General	Nonreasoning	Llama 2 or Vicuna [3]	Vision-language instruction dataset	SFT	7B, 13B
Qwen3-VL-Instruct	General	Nonreasoning	N/A	Vision-language instruction dataset+reasoning data	SFT+Distillation+SAPO^n^ [57]	8B, 30B (3B activated)
Qwen3-VL-Thinking	General	Reasoning	N/A	Vision-language instruction dataset+reasoning data	SFT+Distillation+SAPO [57]	8B, 30B (3B activated)
LLaVA-CoT^o^	General	Reasoning	Llama 3-Vision-Instruct	Reasoning data generated by GPT-4o	SFT	11B
QVQ^p^-Preview	General	Reasoning	Qwen2-VL	Unknown	Unknown	72B
MedGemma-it	Medical	Nonreasoning	MedGemma	Medical image and text data	Unknown	4B, 27B
HuatuoGPT-Vision	Medical	Nonreasoning	LLaVA or Yi [58]	Medical VQA^q^ dataset	SFT	7B, 34B
QoQ-Med	Medical	Reasoning	Qwen2.5-VL	Multimodal (1D, 2D, 3D) medical dataset [56]	DRPO^r^	7B, 32B

a LLM: large language model.

b VLM: vision-language model.

c SFT: supervised fine-tuning.

d RLHF: reinforcement learning from human feedback.

e DPO: direct preference optimization.

f GRPO: group relative policy optimization.

g N/A: not applicable.

h STEM: Science, Technology, Engineering, and Mathematics.

i CoT: chain-of-thought.

j RL: reinforcement learning.

k KTO: Kahneman-Tversky optimization.

l PPO: proximal policy optimization.

m LLaVA: Large Language and Vision Assistant.

n SAPO: soft adaptive policy optimization.

o LLaVA-CoT: Large Language and Vision Assistant–Chain-of-Thought.

p QVQ: Qwen With Vision and Questions.

q VQA: visual question answering.

r DRPO: domain-aware group relative policy optimization.

### Experimental Setting

#### Prompts for Models

In our experiments, we use task-specific prompts tailored to each dataset (Figures S1 and S2 in Multimedia Appendix 1). Consistent with the approach in another study [34], we append a common instruction at the end of each prompt, “return your final response within \boxed{{}}.,” to extract the final answer of the model clearly. Additionally, for models not explicitly designed for reasoning (eg, Llama 3 and Qwen2.5), we explicitly include the phrase “let’s think step by step” at the end of the prompt to encourage step-by-step reasoning by chain-of-thought during inference. An ablation study demonstrating the critical role of reasoning triggers in nonreasoning models is provided in Multimedia Appendix 1 as Figure S7.

#### Test-Time Scaling Across Different Token Budgets

**Figure 2.**
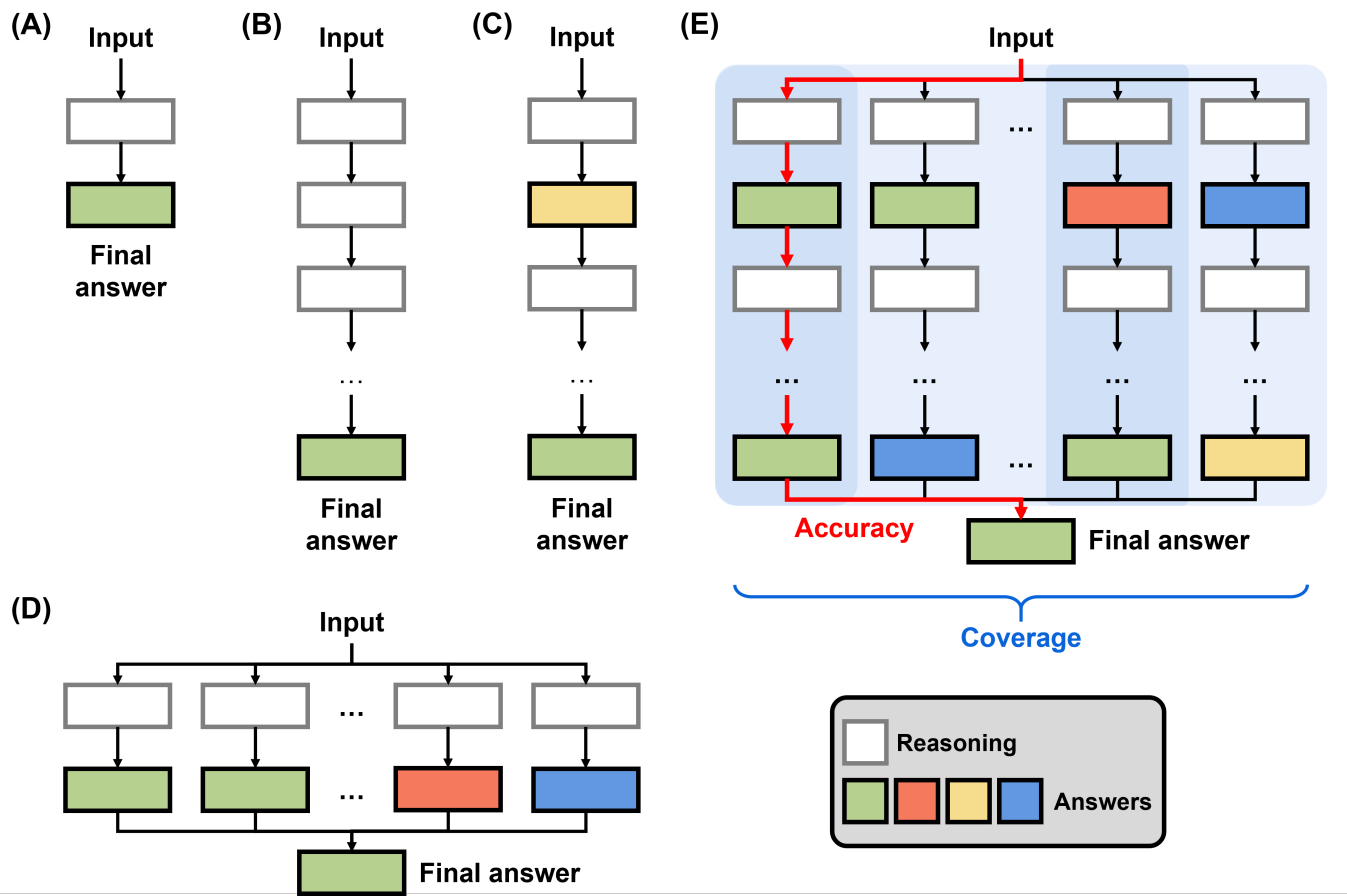
Overview of test-time scaling strategies: (A) no scaling, (B) increasing the token budget, (C) iterative sequential scaling, (D) parallel scaling, and (E) hybrid sequential-parallel scaling.

We investigate test-time scaling in the medical domain by varying the token budget, specifically by adjusting the maximum sequence length allowed for model generation (Figure 2B). If the model reaches this maximum sequence length, its output is truncated accordingly. In cases where the model does not produce a final answer within the allotted length, we append “\boxed{{” to the end of the response and force the model to generate the final answer, which is then used for evaluation. Conversely, if the model completes its reasoning and provides a final response before reaching the token limit, we do not force it to continue generating tokens. To assess how efficiently models use the available token budget, we compute the average number of tokens used during the reasoning process and compare this across models and datasets. We set the temperature to 0 for the token budget experiments.

Next, we evaluate model accuracy across different token budgets. For the medical QA benchmarks, accuracy is measured by the number of final answers that exactly match the ground truth. In contrast, for the MedCalc-Bench dataset, we apply task-specific evaluation criteria: for equation-based calculation problems with decimal answers, a response is considered correct if it falls within a 5% error margin of the correct value; for all other problem types, only exact matches with the correct answer are counted as correct.

#### Sequential and Parallel Scaling

Several studies have compared sequential scaling and parallel scaling in the general domain [18,20], with mixed findings. Motivated by this, we investigate which approach is more effective in the medical domain.

For iterative sequential scaling, responses are not generated all at once within the full token budget. Instead, the model is initially prompted to generate a response within a limited budget (512 tokens). Subsequently, the model is iteratively prompted to revise and extend its previous response. To initiate this revision process, we append the token “wait” at the end of each response, prompting the model to continue and refine its reasoning in subsequent steps, as proposed in previous works [17,34].

In contrast, parallel scaling involves generating multiple responses simultaneously. To determine the final answer from these responses, we apply the shortest majority vote strategy [20]. This strategy computes a specific score for each unique answer category. The score is calculated by dividing the number of solutions in that category by the logarithm of their average length. The final answer is chosen from the category with the highest score. This mathematical formulation addressed a known characteristic of certain reasoning models where performance deteriorates with overly prolonged solution lengths. By penalizing excessive length logarithmically while heavily weighting answer frequency, this approach effectively balances robust consensus with an empirical safeguard against hallucination or error accumulation. To explore the optimal combination of test-time scaling strategies, we additionally experiment with a hybrid approach that integrates both sequential and parallel scaling. This allows us to investigate whether combining iterative refinement with diversity from parallel sampling can lead to improved performance in medical reasoning tasks.

Figure 2A, 2C, and 2D illustrate the concepts of no test-time scaling, sequential and parallel scaling, respectively. To determine whether a model has chosen the correct reasoning path, we extract the conclusion from the generated output and strictly compare it against the ground truth label of the dataset. We evaluate model performance using two metrics: accuracy and coverage. Accuracy measures the model’s success in selecting the correct reasoning path and arriving at the correct conclusion, thereby reflecting its decision efficiency. Coverage, on the other hand, measures the proportion of correct answers found in all intermediate outputs, whether across sequential steps or parallel samples, and captures the model’s capacity to explore a diverse reasoning space.

A model with high coverage but low final accuracy suggests it can generate valid reasoning paths but often fails to select them as the final answer. This implies strong exploratory capacity but a tendency to diverge from correct conclusions due to overthinking, inconsistent logic, or ineffective selection strategies.

To control generation variability, we set the temperature to 0 for sequential scaling and 0.7 for parallel scaling.

#### User-Driven Factor

We also investigate whether test-time scaling enhances the robustness of LLMs to user-driven factors using three medical QA benchmark datasets: MedQA, Medbullets, and MedXpertQA. Building on prior work [45,59,60], we focus on scenarios where the user injects misleading information into the prompt.

In these experiments, we insert misleading hints into the prompt, presented as another physician’s opinion favoring an incorrect answer. To maximize confusion, we use GPT-4o to select the most semantically plausible incorrect option. We then incorporate this misleading hint into the prompt, varying two factors: the tone of the comment (hedged vs definitive) and the expertise level of the physician (novice vs expert). The following examples illustrate the different conditions: (1) hedged tone, novice physician: “Comment from another novice physician: I think the answer is probably A.” (2) Hedged tone, expert physician: “Comment from another expert physician: I think the answer is probably A.” (3) Definitive tone, novice physician: “Comment from another novice physician: I am confident the answer is A.” (4) Definitive tone, expert physician: “Comment from another expert physician: I am confident the answer is A.”

### Ethical Considerations

This study does not involve human participants, animal participants, medical records, or any personally identifiable information. All experiments were conducted exclusively using publicly available and anonymized medical benchmark datasets to evaluate LLMs. Therefore, this study does not constitute human participants research and did not require formal review or approval by an institutional review board or a local ethics committee, in accordance with standard institutional and national policies regarding the secondary analysis of publicly available data.

## Results

### Test-Time Scaling Across Different Token Budgets

#### Experiments With LLMs

**Figure 3.**
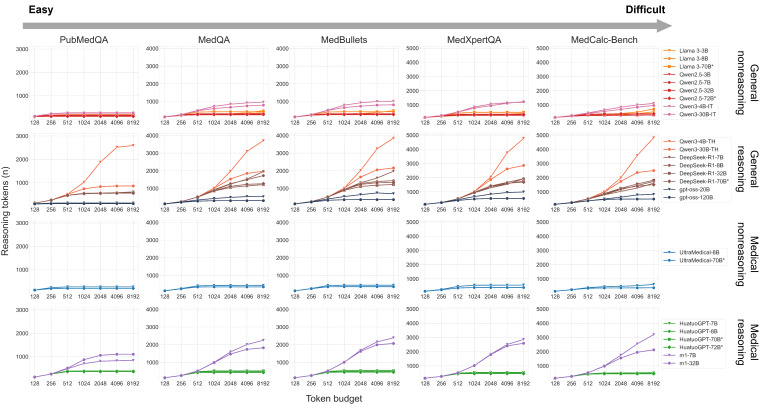
Test-time scaling of various large language models across different token budgets: average number of reasoning tokens used. * in the legend indicates models that are 4-bit quantized.

**Figure 4.**
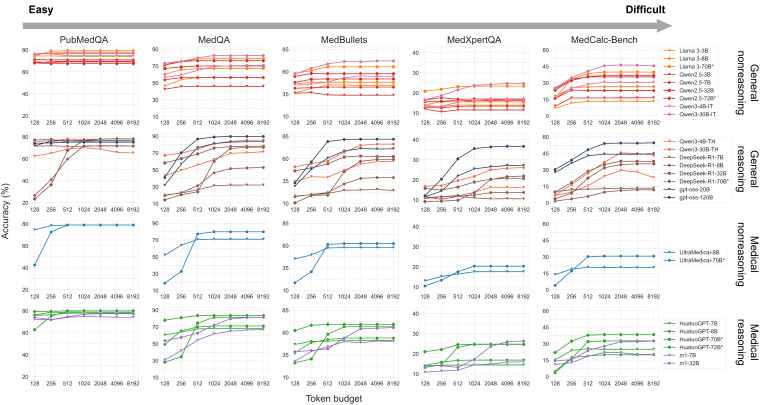
Test-time scaling of various LLMs (large language models) across different token budgets: accuracy of LLMs as a function of token budget. * in the legend indicates models that are 4-bit quantized.

First, we investigate whether test-time scaling of LLMs is effective in the medical domain by increasing the token budget. Figure 3 presents the average number of reasoning tokens used under different token budgets, where the first row displays Llama-based LLMs and the second row shows Qwen-based LLMs. Figure 4 presents the accuracy of various models across different token budgets.

Regarding nonreasoning models, as illustrated in Figure 3, LLMs that lack explicit reasoning capabilities typically use only a small portion of the available token budget (approximately 500 to 1000 tokens), even when a larger budget is provided. Consequently, as shown in Figure 4, their accuracy quickly saturates and does not improve with increased token budgets. This effect is especially evident in smaller models with fewer than 10B parameters. These observations suggest that simply increasing the token budget at test time is unlikely to enhance performance for intrinsic nonreasoning models.

For reasoning models, most show minimal or no improvement in performance on the easier task (PubMedQA) as the token budget increases. However, as the task difficulty increases (defined in the Methods section), reasoning models tend to use more reasoning tokens. Notably, the degree and manner of this token usage vary across different reasoning models, each exhibiting distinct trends in response to increasing task complexity.

For example, DeepSeek-R1 or Qwen3-TH models tend to use more tokens for reasoning as the token budget increases, and perform better with increased token budgets. This effect is more pronounced in models with larger parameter counts. However, the smaller variants of DeepSeek-R1 (7B or 8B) perform worse than other general-domain models of similar size. We hypothesize that this is because the versions of DeepSeek-R1 used in our experiments are distilled models trained on synthetic data (generated by the original DeepSeek-R1) using SFT, rather than through RL. As a result, these smaller distilled models may fail to fully retain the reasoning capabilities of the original model, leading to their lower performance.

The gpt-oss models demonstrate adaptive token usage based on task complexity. On simpler benchmarks such as PubMedQA and MedQA, these models maintain relatively low token usage even when provided with large budgets, suggesting that extensive reasoning is not triggered for easier queries. However, as task difficulty increases, the models exhibit an upward trend in token consumption, using a large portion of the available budget. Crucially, this increased usage of reasoning tokens correlates with performance gains. The gpt-oss models achieve higher accuracy on challenging tasks as the token budget expands. To further illustrate this direct relationship, we provide an explicit plot of actual token consumption against accuracy for all evaluated models in Figure S6 in Multimedia Appendix 1. This visualization confirms that for reasoning-capable models, increased token usage is strongly correlated with improved accuracy, particularly on complex tasks.

HuatuoGPT-o1 generally exhibits strong performance across all experiments. However, despite being categorized as a reasoning model, it tends to use a relatively small number of tokens during its reasoning process. As a result, it demonstrates limited performance gains from test-time scaling via increased token budgets compared to other reasoning models.

In contrast, m1 effectively uses a larger portion of the available token budget and exhibits substantial performance improvements as the token budget increases. Notably, on the most challenging QA dataset in our study, MedXpertQA, m1-32B demonstrates clear test-time scalability, outperforming other models with even larger parameter counts as the token budget increases. We attribute the difference between HuatuoGPT-o1 and m1 to differences in the nature of their respective training data. While the reasoning traces used to train HuatuoGPT-o1 were generated by GPT-4o [6,29], those used for m1 were synthesized by DeepSeek-R1 [9,34]. These underlying models differ in their reasoning styles and output structures, likely leading to variations in the length and structure of chain-of-thought traces and influencing how each model uses tokens during inference.

Regarding general vs medical models, as expected, medical LLMs generally outperform general domain LLMs on medical QA tasks, except for the recently released Qwen3 variants and gpt-oss. However, in the case of MedCalc-Bench, the accuracy of medical models is generally lower than that of general domain models. This discrepancy suggests that MedCalc-Bench primarily rewards general numerical or procedural reasoning applied within a medical context, rather than the qualitative clinical reasoning that centralizes specialized medical training. Therefore, the stronger performance of general-purpose models does not necessarily imply deficient medical reasoning overall. Instead, it indicates that the benchmark captures a narrower procedural capability that is less tied to domain-specific clinical expertise. Nevertheless, performance can still be improved through test-time scaling with an increased token budget.

In summary, test-time scaling through increased token budgets can improve performance for certain reasoning LLMs. However, many existing LLMs that benefit from this approach are not specifically designed for the medical domain. This highlights the need to explore alternative test-time scaling strategies better suited for medical applications.

#### Experiments With VLMs

Next, we investigate the test-time scaling in the medical domain using VLMs. Similar to the previous experiment, we analyze the impact of increasing token budgets on model performance.

**Figure 5.**
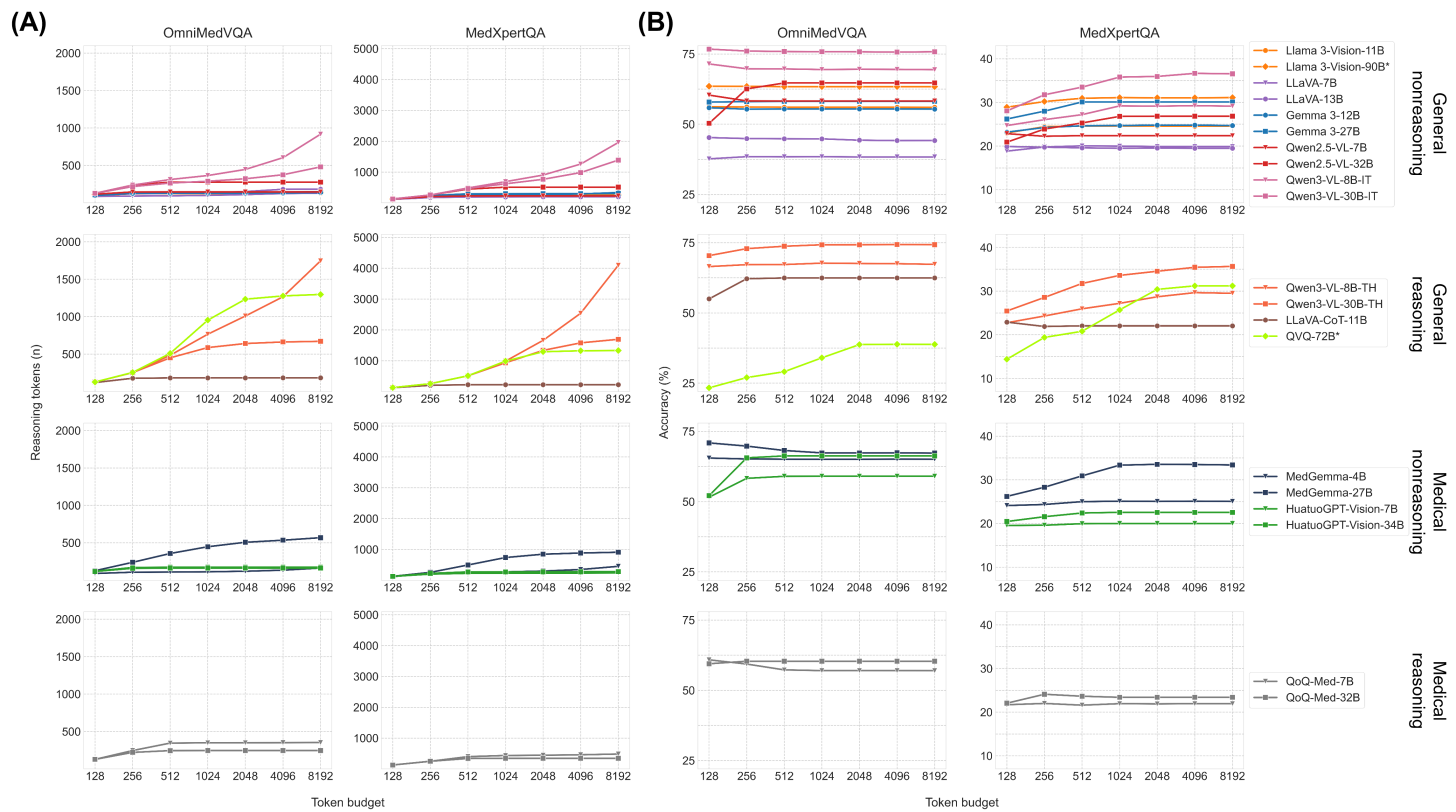
Test-time scaling of VLMs (vision-language model) across medical VQA (visual question answering) benchmark: (A) average number of reasoning tokens used and (B) accuracy of LLMs (large language model) as a function of token budget. * in the legend indicates models that are 4-bit quantized.

Figure 5A shows the average number of tokens used by VLMs during reasoning. Consistent with our findings from LLMs, most models do not significantly increase their token usage, even when given a higher token budget. In contrast, models such as the Qwen3-VL variants, MedGemma-27B, and QVQ exhibit a distinct upward trend in token usage as the available token budget increases, indicating their capacity to leverage additional computational resources for extended reasoning.

Figure 5B presents model accuracy on two datasets across different token budgets. On OmniMedVQA, which is relatively simple and less demanding in reasoning, most models do not show any performance improvement as the token budget increases. In contrast, on MedXpertQA, a dataset requiring more complex reasoning, we observe a more noticeable, though still limited, test-time scaling effect. Conversely, models that exhibit an upward trend in token usage demonstrate clear performance gains from increased token budgets on MedXpertQA, a correlation that is particularly pronounced in large-scale models with 27B or more parameters.

Interestingly, while QVQ displays test-time scaling on both benchmarks, its performance is paradoxically lower on the simpler OmniMedVQA and higher on the more challenging MedXpertQA. Upon closer inspection of QVQ’s responses, we attribute this to a combination of model limitations and dataset characteristics. Specifically, QVQ frequently fails to process the input images in OmniMedVQA correctly. It sometimes outputs messages such as “I can’t see the image” (Figure S3 in Multimedia Appendix 1) or misinterprets prompts as part of the image content (eg, “There are also some text elements overlaid on the image, such as ‘You are a helpful assistant’”; Figure S4 in Multimedia Appendix 1). Additionally, due to these misinterpretations, QVQ often fails to choose from the provided answer options, instead responding with “none of the above” (Figure S5 in Multimedia Appendix 1), further reducing its accuracy. In contrast, the performance of QVQ on MedXpertQA benefits from the presence of more informative textual clues within the questions, while the questions of OmniMedVQA are very simple (Figure S2 in Multimedia Appendix 1). These clues often enable the model to infer the correct answer even without fully using the image, thus reducing the impact of its visual processing limitations. As a result, QVQ achieves higher accuracy despite the greater task complexity.

Overall, our findings from the VLM experiments are consistent with those from the LLM experiments: test-time scaling, by simply increasing the token budget, provides limited benefits for most VLMs. This approach shows effectiveness only for some models, and even then, the performance gains in VLMs are considerably smaller than those observed in LLMs. Notably, as in the case of LLMs, the effectiveness of test-time scaling in VLMs is more pronounced on more difficult tasks, while it has little to no impact on simpler tasks.

These results can be interpreted from two perspectives. First, unlike LLMs, most of the current VLMs may lack sufficiently developed reasoning capabilities, particularly in leveraging visual cues for complex decision-making. While recent studies have proposed VLMs trained for reasoning, these models are often not explicitly trained to reason effectively using visual information and thus struggle to interpret and analyze complex medical images as effectively as LLMs do with text. However, our findings point to potential pathways for enhancing VLM reasoning capabilities. First, the adoption of advanced posttraining methodologies can significantly reinforce reasoning performance. For instance, the Qwen3-VL models were posttrained using SFT, distillation, and Soft Adaptive Policy Optimization [57], an RL method specifically proposed for Qwen3-VL. Consequently, these models exhibit a distinct test-time scaling effect on the challenging MedXpertQS benchmark as the token budget increases. Second, scaling model parameters in conjunction with medical domain training proves beneficial. In our experiment, MedGemma-27B demonstrates improved performance on MedXpertQA as the token budget expands, whereas the smaller MedGemma-4B does not.

Next, the limitations may stem from the current vision-language medical benchmarks, which may not be well-suited for evaluating reasoning ability. For instance, OmniMedVQA primarily consists of relatively simple image-based questions that require minimal reasoning, thus failing to challenge the model’s reasoning capabilities. Conversely, MedXpertQA presents highly challenging questions that often require the simultaneous analysis of multiple medical images. Most contemporary VLMs still struggle with or entirely lack the ability to jointly and effectively reason over such complex visual cues in conjunction with textual information, which may explain the relatively limited performance on this benchmark compared to that of LLMs. Future work should aim to develop stronger medical reasoning VLMs and more rigorous benchmarks for their evaluation.

### Sequential and Parallel Scaling

In previous experiments, we observed that test-time scaling, by simply increasing the token budget, is ineffective for many models. Except for certain reasoning models, most do not use the full available token budget during inference. To address this limitation, we investigate alternative scaling strategies, iterative sequential scaling and parallel scaling, as described in the Methods section. In this experiment, we evaluate two medical reasoning LLMs: HuatuoGPT-o1-7B (denoted as HuatuoGPT-7B) and m1-7B-1k (denoted as m1-7B).

**Figure 6.**
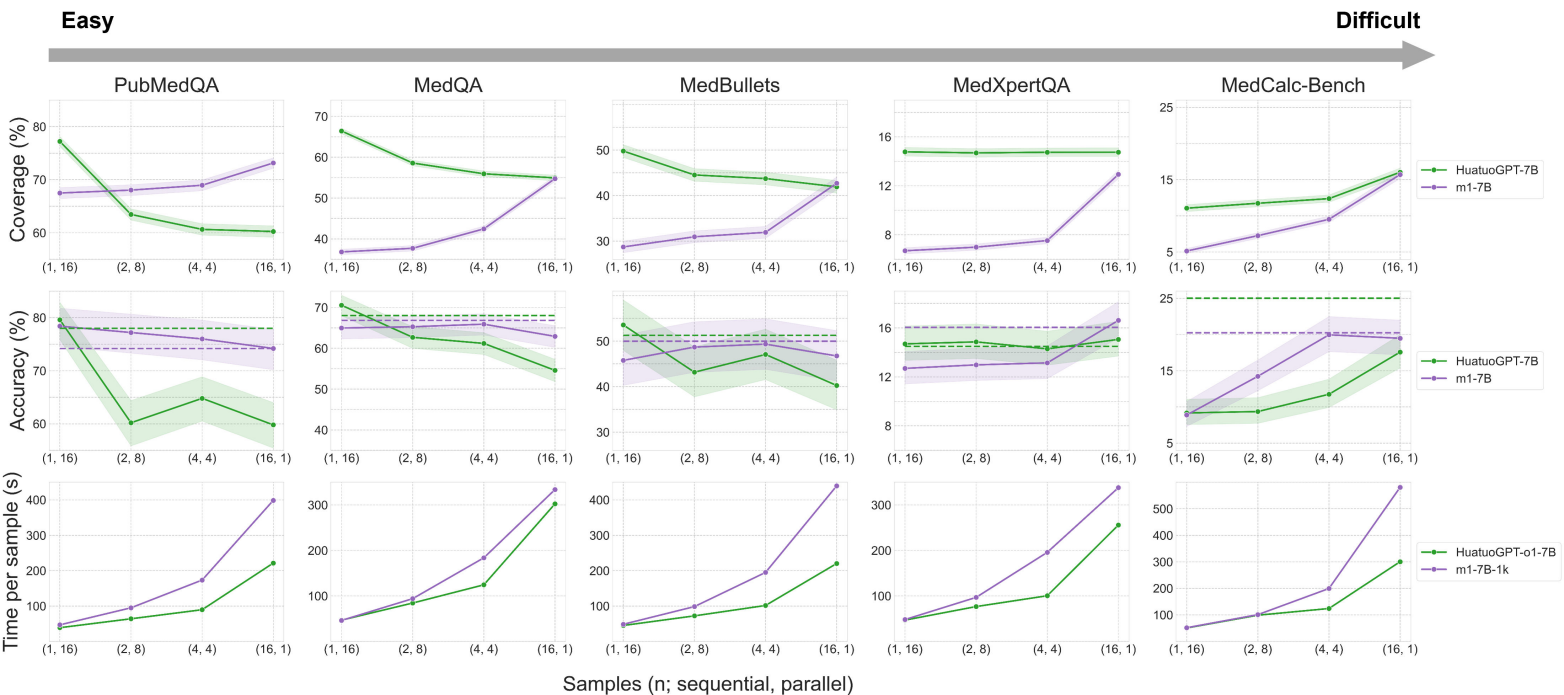
Comparison of sequential and parallel test-time scaling of LLMs (large language models) across medical benchmarks. Dotted lines indicate accuracy with an increased token budget to (8192). Shaded regions represent 95% CIs.

Figure 6 presents a comparison between sequential and parallel test-time scaling across multiple medical benchmarks. On PubMedQA, the easiest QA task, both models show a decline in accuracy as the number of sequential sampling steps increases. Moreover, the accuracy achieved with parallel scaling is higher than that obtained by simply increasing the token budget. This suggests that additional iterations may not be beneficial for simpler tasks; in fact, excessive revisions can lead the models to deviate from initially correct answers. In particular, for m1, accuracy decreases even though coverage increases with more sequential steps, indicating that while the model may identify the correct answer during sequential scaling, unnecessary revisions can lead to an incorrect final output.

For MedQA and MedBullets, HuatuoGPT-o1 maintains the same trend, showing limited improvement from either sequential scaling or increased token budgets. In contrast, m1 achieves its highest accuracy when the token budget is evenly divided between sequential and parallel sampling (1:1 ratio), with performance comparable to that of simple token budget expansion. These findings suggest that for tasks of moderate difficulty, sequential scaling may offer little advantage for models that naturally generate shorter outputs. For these models, the performance gains from parallel scaling on simpler tasks are driven primarily by reasoning path diversity and answer consistency through majority voting, rather than the brevity of the reasoning itself. In comparison, models such as m1, which rely on deeper reasoning, benefit more from a balanced approach that combines diverse generation with iterative refinement.

On the more challenging MedXpertQA benchmark, which demands complex reasoning, HuatuoGPT-o1 shows relatively consistent performance across all scaling strategies, including increased token budget, sequential scaling, and parallel scaling. In contrast, m1 achieves its highest accuracy and coverage with fully sequential scaling, slightly surpassing its performance under the increased token budget condition.

On MedCalc-Bench, which also involves complex reasoning but with distinct characteristics, both models show improved performance as the number of sequential samples increases. However, the highest accuracy is attained when applying test-time scaling via a larger token budget. These findings suggest that step-by-step sequential refinement benefits reasoning-intensive question-answering tasks, while generating a single, extended reasoning path is more effective for tasks requiring precise calculations.

In summary, under constrained token budgets, parallel scaling proves highly effective for simpler tasks. This advantage stems from the model exploring a diverse set of reasoning paths and arriving at a reliable conclusion through answer consistency. In contrast, for models that rely on more extensive reasoning, sequential scaling or increasing the token budget becomes increasingly advantageous as task complexity grows.

In addition to evaluating task performance, we analyzed the computational cost, specifically the inference latency (wall-clock time), associated with each scaling strategy to assess their viability for clinical deployment. As illustrated in the bottom row of Figure 6, increasing the proportion of sequential reasoning results in a substantial increase in processing time per sample. This delay occurs because sequential scaling relies on iterative generation, where each step must wait for the previous one to complete, while the context length continuously expands. Conversely, heavily parallelized configurations maintain significantly lower latency. By using batched inference, parallel scaling processes multiple samples simultaneously without a proportional increase in wall-clock time. This quantitative analysis reveals a critical cost-performance trade-off, indicating that parallel scaling is highly efficient for time-sensitive clinical applications.

### Impact of Test-Time Scaling on User-Driven Factors

**Figure 7.**
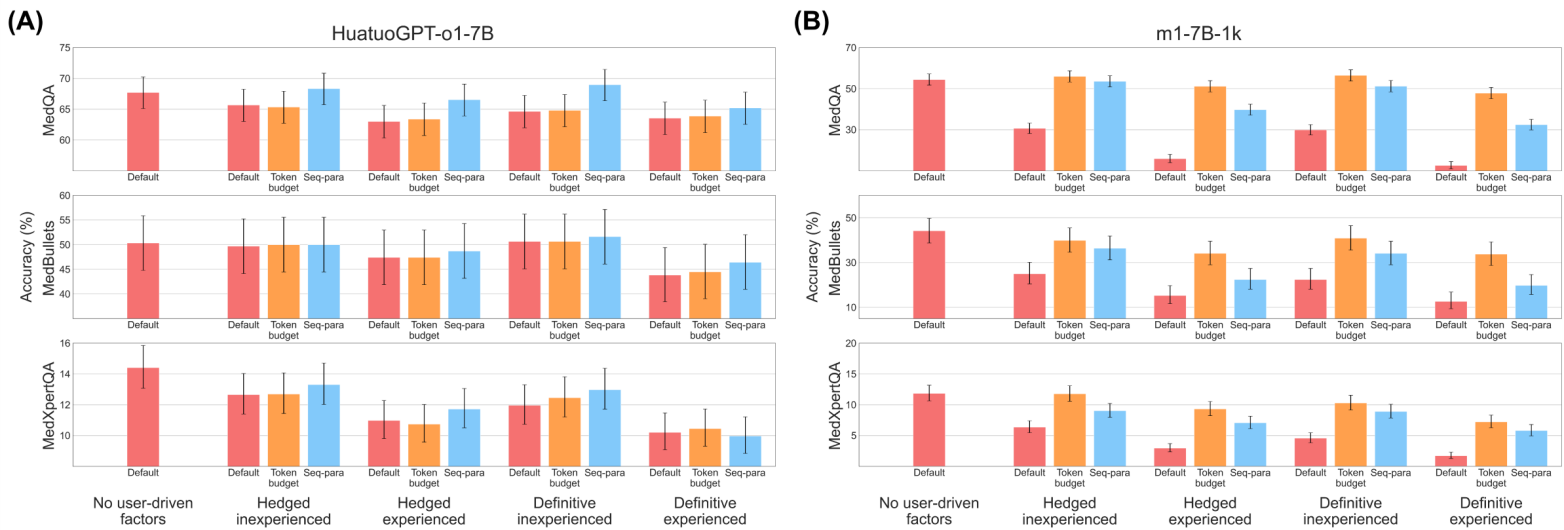
Test-time scaling under different user-driven factor types on medical QA (question answering) benchmarks. (A) HuatuoGPT-o1-7B and (B) m1-7B-1k. Error bars represent 95% CIs.

Finally, we investigate the impact of test-time scaling on robustness to user-driven factors. Again, we evaluate two medical LLMs, HuatuoGPT-o1-7B and m1-7B-1k, across two types of test-time scaling strategies. The first strategy simply increases the token budget by a factor of 16 from the default setting, as described in the first experiment. The second strategy combines iterative sequential scaling and parallel scaling using the optimal configuration identified in the second experiment. Figure 7 shows the accuracy of both models under various user-driven perturbations and test-time scaling strategies.

First, Figure 7A shows that user-driven perturbation generally degrades the performance of HuatuoGPT-o1 when no test-time scaling strategy is applied. When test-time scaling is applied by simply increasing the token budget, the improvement is minimal, and in some cases, performance even declines. In contrast, applying the optimal test-time scaling strategy significantly improves robustness against user-driven factors in most scenarios. The benefit is particularly pronounced when the task is easier, such as in MedQA. This is likely because HuatuoGPT-o1 tends to generate relatively short responses by default, and merely increasing the token budget does not effectively encourage the model to use the additional tokens for deeper reasoning.

On the other hand, m1 also exhibits degraded performance in the presence of user-driven factors, as illustrated in Figure 7B. However, unlike HuatuoGPT-o1, increasing the token budget proves more effective than applying the optimal sequential-parallel scaling strategy for the m1 model. This is due to m1’s inherent tendency to generate long chain-of-thought reasoning when solving problems. As sequential-parallel scaling produces shorter responses at each iteration, it may hinder m1 from fully developing its reasoning and reaching a well-considered conclusion.

In summary, model behavior varies depending on the chosen test-time scaling strategy when user-driven factors exist. This highlights the importance of selecting an appropriate test-time scaling method tailored to each model type to enhance robustness against such perturbations. Nonetheless, some common patterns emerge across models. For relatively easy tasks (eg, MedQA), both models can recover performance comparable to that without user-driven interference through test-time scaling. In contrast, for more challenging tasks (eg, MedXpertQA), the performance gap between conditions with and without user-driven factors remains substantial, even when test-time scaling is applied. Notably, models are particularly sensitive to the expertise level of the physician providing the additional input. This suggests that as task complexity increases, LLMs may struggle to initiate reasoning with high confidence, leading them to heavily rely on the initial context or cues provided by the user. Consequently, when such cues are misleading, the model is more likely to follow an incorrect reasoning path and, due to the increased complexity, has greater difficulty recovering and arriving at the correct answer. These findings emphasize the amplified impact of user-driven perturbations in difficult scenarios and highlight the importance of minimizing such perturbations by providing only neutral and essential information when prompting LLMs for complex or high-stakes medical QA tasks.

## Discussion

### Principal Results

To contextualize our findings, the primary aim of this study was to establish a domain-specific framework for scaling inference compute in medical AI. We hypothesized that the optimal scaling strategy depends heavily on the reasoning capacity of the model and the complexity of the clinical task. Our overall results broadly confirm these hypotheses. We demonstrated that simply increasing the computational budget during inference is not a universal remedy. Instead, the efficacy of test-time scaling is highly conditional, varying significantly across model architectures, task difficulties, and clinical contexts.

First, we found that simply increasing the token budget is not a universal solution for enhancing medical LLM or VLM performance. A distinct contrast exists between nonreasoning and reasoning models. Nonreasoning models (eg, Llama 3 and Qwen2.5) exhibited performance saturation, using fewer than 1000 tokens even when provided with larger budgets, resulting in negligible accuracy gains. In contrast, reasoning-optimized models demonstrated adaptive token usage. Notably, the gpt-oss models exhibited behavior aligned with “thinking-optimal” scaling. They maintained efficient, low token usage for simpler tasks such as PubMedQA but increased consumption for complex benchmarks such as MedXpertQA, which correlated directly with accuracy improvements. Furthermore, the efficiency of this scaling appears to be heavily influenced by the nature of the model’s training data. The m1 model, trained on DeepSeek-R1 traces, used more tokens for deep reasoning than HuatuoGPT-o1, which relied on GPT-4o traces.

Second, test-time scaling for VLMs currently lags behind that of LLMs. Most VLMs failed to leverage increased budgets effectively and showed limited improvements in reasoning depth. Exceptions to this trend were observed in Qwen3-VL variants and MedGemma-27B. This suggests that advanced posttraining methods such as Soft Adaptive Policy Optimization and larger parameter sizes are critical enablers for multimodal test-time scaling. Specifically, we observed that scaling model parameters in conjunction with medical domain training proves beneficial.

Third, we demonstrated that the optimal scaling strategy is highly task-dependent. For simpler tasks, excessively long reasoning paths are not necessarily beneficial. Iterative sequential scaling proved harmful on easier datasets such as PubMedQA. This performance drop is likely due to compounding errors during unnecessarily extended reasoning. In this case, parallel scaling using majority voting yielded superior results. This success arises from exploring a diverse set of reasoning paths and aggregating them through majority voting to ensure answer consistency. Conversely, reasoning-intensive tasks such as MedXpertQA and calculation tasks such as MedCalc-Bench benefited from extended sequential reasoning.

Finally, while test-time scaling enhances robustness against use-driven perturbations, it requires model-specific tailoring. We observed that scaling strategies generally mitigated the negative impact of misleading expert hints but often failed to fully restore baseline performance on difficult tasks. Crucially, the effective strategy varied by model architecture. HuatuoGPT-o1 benefited most from a hybrid sequential-parallel approach. However, m1 relies on generating long, uninterrupted reasoning chains and performs best with a simple expansion of the token budget. This underscores the necessity of aligning scaling strategies with both the difficulty of the clinical task and the intrinsic reasoning behaviors of the specific model being deployed.

### Comparison With Prior Work

Prior studies on test-time scaling in general domains, such as mathematics and coding, have established that increasing inference compute can improve reasoning, though the relationship is not strictly monotonic. These works demonstrate that optimal reasoning lengths exist and that excessive self-revision can sometimes degrade performance [18-20]. While these foundational works highlight the need for task-dependent scaling strategies, our study reveals that in the high-stakes medical domain, the limitations of test-time scaling go beyond mere overthinking and manifest through three unique, domain-specific bottlenecks.

First, text-centric scaling limits encounter a fundamentally different bottleneck in multimodal clinical environments. While prior work focuses on finding the optimal token length to balance reasoning depth and diversity, our evaluation of VLMs shows that the issue is not about optimal length but rather a structural inability to integrate complex visual clues. Simple token expansion fails to trigger meaningful multimodal reasoning, indicating that safe medical AI deployment requires fundamental vision-language alignment improvements rather than just optimizing computational scaling.

Second, we identify a domain-specific disparity in scaling efficiency between qualitative clinical and quantitative procedural logic. Unlike general studies that evaluate scalability based on parameter size or general reasoning datasets, we demonstrate that medically fine-tuned LLMs excel in text-based medical QA but exhibit degraded scaling efficiency on clinical calculation tasks compared to general-domain models. This disparity does not necessarily imply that medical fine-tuning inherently impairs reasoning capabilities. Instead, it suggests that current medical alignment heavily prioritizes qualitative clinical knowledge over the general numerical and procedural reasoning captured by calculation benchmarks. Consequently, test-time scaling strategies behave inconsistently depending on whether the clinical task relies on deep domain-specific expertise or general numerical computation, an insight unique to the medical domain.

Third, our work uncovers a unique cognitive vulnerability to perceived clinical authority. Standard robustness evaluations in prior test-time scaling typically focus on a model’s ability to self-correct against random noise or generic misleading logic. In contrast, we demonstrate that when models are confronted with deceptive hints framed specifically as expert physician opinions, they readily abandon their correct reasoning pathways. Under this hierarchical clinical pressure, even optimal scaling strategies fail to restore baseline performance, which represents a distinct vulnerability that differentiates our work from general-domain robustness findings.

### Practical Considerations for Clinical Deployment

While our experimental design controls for the token budget to evaluate the cognitive limits of reasoning length, it is important to acknowledge that token count does not equate to equal computational cost across different model architectures. Generating the same number of tokens with a large-scale model consumes substantially more compute (floating point operations per second), energy, and financial resources than a smaller model. Therefore, evaluating deployment feasibility requires balancing reasoning performance against strict computational constraints.

Furthermore, different test-time scaling strategies exhibit distinct cost-performance trade-offs. As observed in our experiments, parallel scaling can be implemented efficiently using batched inference, where model weights are loaded only once. This minimizes the increase in GPU memory footprint to just the required key-value cache and significantly reduces wall-clock time, making it highly suitable for time-sensitive environments such as emergency departments. Conversely, sequential scaling inherently increases latency due to its serial nature and expanding context length, limiting its use to nonurgent, complex diagnostic scenarios. Ultimately, health care institutions must carefully weigh these factors. They should choose whether to deploy a massive model or to apply efficient parallel scaling on a smaller, medically aligned model based on their specific latency requirements and computational budgets.

### Limitations

Although we conducted a broad investigation of test-time scaling in the medical domain, several limitations remain. First, our study focused on scaling strategies such as increasing the token budget, iterative self-revision via sequential scaling, and generating multiple responses in parallel. However, other promising strategies, such as using trained verifiers to select the most accurate response among candidates, were not explored. In addition, compared to LLMs, VLMs require more comprehensive evaluation under test-time scaling. While our results show that test-time scaling can improve robustness to user-driven factors, it was not sufficient to fully restore performance to baseline levels observed without such perturbations. Future work should investigate more advanced or hybrid test-time scaling techniques that can further mitigate the impact of user-driven factors. Finally, our experiments were limited to open-source LLMs, while proprietary models such as GPT (OpenAI), Claude (Anthropic, PBC), or Gemini (Google LLC) were not included. Extending test-time scaling evaluations to these models represents an important direction for future research in the medical domain.

A notable limitation of our study is that the evaluation relies on specific benchmark datasets, which serve as finite samples of the much broader population of real-world medical questions. Consequently, the reported accuracy metrics carry inherent statistical uncertainty. In scenarios where performance differences across models or scaling strategies are marginal, identifying a single optimal model based solely on empirical accuracy may not fully reflect their true generalization ability. Future research evaluating LLMs in health care should incorporate rigorous statistical frameworks to quantify this uncertainty. Using methodologies to construct confidence sets of potential best performers [61-63] would provide a more robust and statistically sound approach to model selection when a clear single winner is not evident.

Additionally, as with most current LLM evaluations, we cannot completely rule out the possibility of data contamination, where public benchmark questions might have been included in the base models’ pretraining corpora. However, the core focus of this study is the relative performance gain achieved through test-time scaling rather than absolute baseline accuracy. If models were merely retrieving memorized answers, increasing the token budget or applying sequential scaling would not yield the significant accuracy improvements we observed. Furthermore, our perturbation experiments using user-driven factors demonstrate that models engage in active, dynamic reasoning rather than static memory retrieval. Nonetheless, future studies using completely private or recently curated clinical datasets will be essential to further validate the extent of test-time scaling benefits free from any potential memorization bias.

### Conclusions

In this paper, we conducted a comprehensive investigation of test-time scaling in the medical domain across various types of LLMs and VLMs. Our experiments demonstrate that longer reasoning is not universally beneficial across all medical tasks. For simpler tasks, extensive reasoning is unnecessary, and models that produce concise reasoning traces offer better computational efficiency. In contrast, for complex and challenging tasks, generating longer chain-of-thought reasoning leads to better performance, with models capable of producing extended reasoning traces outperforming their counterparts. Additionally, we observed that medical LLMs tend to perform worse on medical calculation tasks compared to general domain LLMs. This trend likely reflects that current medical fine-tuning practices heavily focus on qualitative textual medical knowledge, meaning these models are less optimized for the procedural, calculation-heavy demands captured by such benchmarks rather than enduring an actual impairment of reasoning capabilities.

Furthermore, we examined whether sequential or parallel test-time scaling provides greater benefits. Our findings indicate that the optimal test-time scaling strategy varies depending on both the model type and the difficulty of the task. Additionally, we found that selecting an appropriate scaling strategy is crucial for enhancing model robustness against user-driven factors, such as misleading or biased information in the prompts. Notably, the impact of these user-driven factors becomes more pronounced as task difficulty increases, emphasizing the importance of minimizing perturbations by providing only neutral and essential information for complex tasks. A summary of our findings is presented in Tables 3 and 4.

Beyond benchmark performance, these findings hold broader implications for the real-world deployment of medical AI. As health care institutions increasingly integrate AI into clinical workflows, treating inference compute as a static resource is no longer viable or safe. Our study highlights that ensuring patient safety, diagnostic accuracy, and operational efficiency requires a dynamic approach to model deployment. Computational strategies must be carefully tailored to the specific urgency and complexity of the medical scenario. Ultimately, understanding when and how to appropriately scale reasoning at test time is a crucial foundational step toward developing clinical AI systems that are not only computationally efficient but also robust, resilient, and trustworthy in high-stakes health care environments.

**Table 3.** Recommended test-time scaling strategies based on task difficulty in the medical domain.

Difficulty	Recommendation
Easy	Prefer parallel scaling over simply increasing the token budget or using iterative sequential scaling Avoid user-driven factors that use a definitive tone and reflect expert-level input
Intermediate	Adapt the scaling strategy based on the model’s token usage pattern, combining sequential and parallel scaling as appropriate Avoid user-driven factors that use a definitive tone
Difficult	Apply iterative sequential scaling for question-answering tasks to enhance step-by-step reasoning Increase the token budget for calculation tasks that require extended reasoning Avoid all types of user-driven factors and provide only neutral and essential information

**Table 4.** Recommended test-time scaling based on model types in the medical domain.

Model type	Strength	Weakness	Recommendation
General, nonreasoning	Performs well on medical calculation tasks	Limited medical knowledge Relatively low performance on reasoning-intensive medical question answering tasks Use a small number of tokens for reasoning	Use primarily for medical calculation tasks Prefer parallel scaling over other strategies
General, reasoning	Performs well on medical calculation tasks High performance on reasoning-intensive tasks	Limited medical knowledge Relatively low performance on reasoning-intensive medical question answering tasks Underperform when using small model sizes	Use primarily for medical calculation tasks Adjust scaling strategy based on the model’s token usage pattern and task difficulty, combining sequential and parallel scaling as needed Use sufficiently large models (over 30B parameters)
Medical, nonreasoning	Performs well on medical question-answering tasks	Relatively low performance on reasoning-intensive medical calculation tasks Use a small number of tokens for reasoning	Use primarily for medical question answering tasks Prefer parallel scaling over other strategies
Medical, reasoning	Performs well on medical question-answering tasks High performance on reasoning-intensive tasks	Relatively low performance on reasoning-intensive medical calculation tasks	Use primarily for medical question answering tasks Adjust scaling strategy based on the model’s token usage pattern and task difficulty, combining sequential and parallel scaling as needed

## Supplementary material

10.2196/90693 Multimedia Appendix 1 Prompts for large language models or vision-language models, examples of responses of QVQ (Qwen With Vision and Questions), and results of ablation studies.

## Abbreviations

CLIMB: clinical large-scale integrative multimodal benchmark
ECG: electrocardiogram
LLaVA: Large Language and Vision Assistant
LLaVA-CoT: Large Language and Vision Assistant–Chain-of-Thought
LLM: large language model
QA: question answering
QVQ: Qwen With Vision and Questions
RL: reinforcement learning
SFT: supervised fine-tuning
USMLE: United States Medical Licensing Examination
VLM: vision-language model
VQA: visual question answering
