# Supplementary material for: Model and Task-Aware Test-Time Scaling Strategies for Large Language and Vision-Language Models in Medicine: Evaluation Study
Source: J Med Internet Res. 2026 Jul 23;28:e90693. doi: 10.2196/90693 (PMC13397005; doi:10.2196/90693)
Supplement: Multimedia Appendix 1 — Prompts for large language models or vision-language models, examples of responses of QVQ (Qwen With Vision and Questions), and results of ablation studies. [file jmir-v28-e90693-s001.docx]

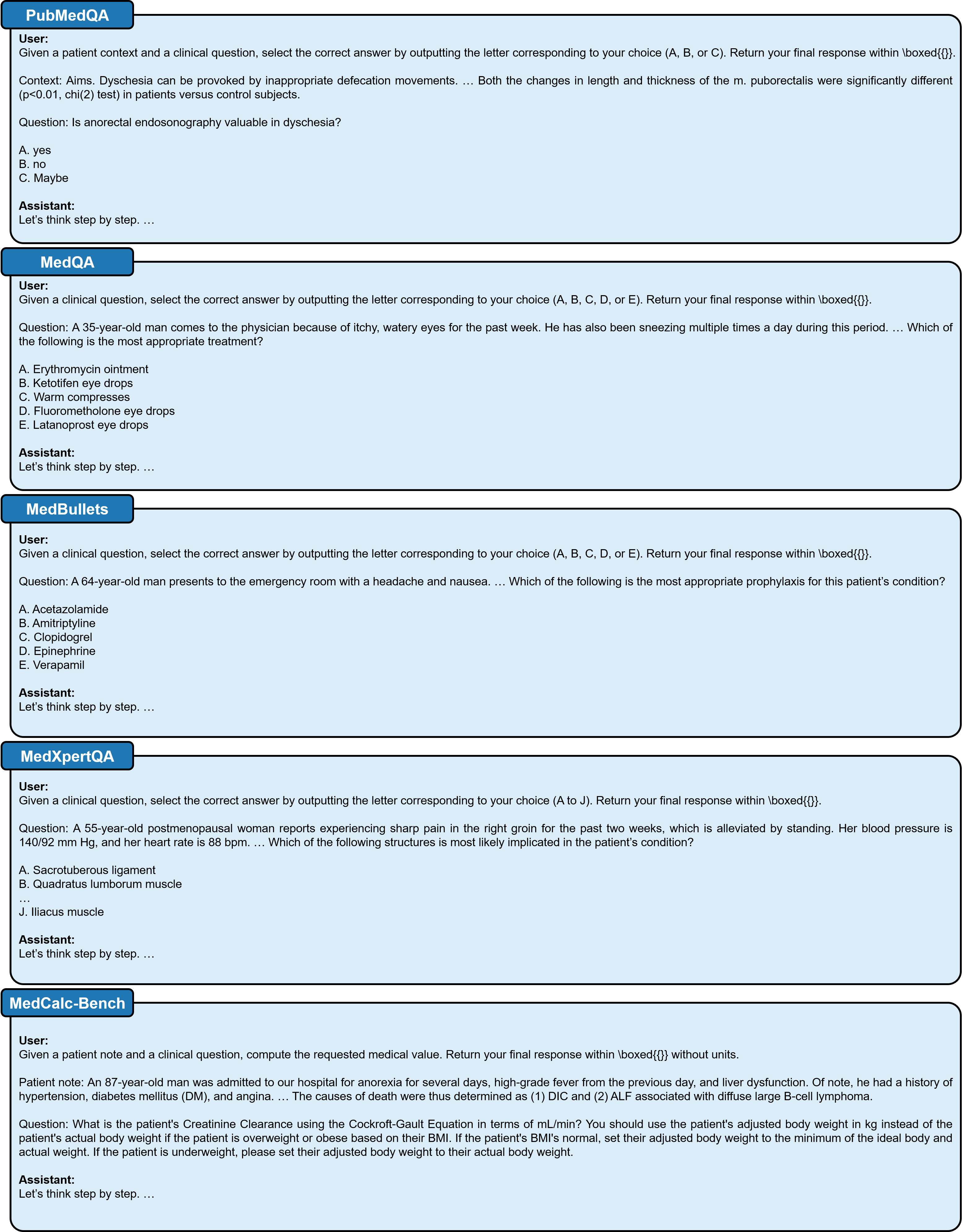


Figure S1. Prompts for text-only medical benchmark datasets.


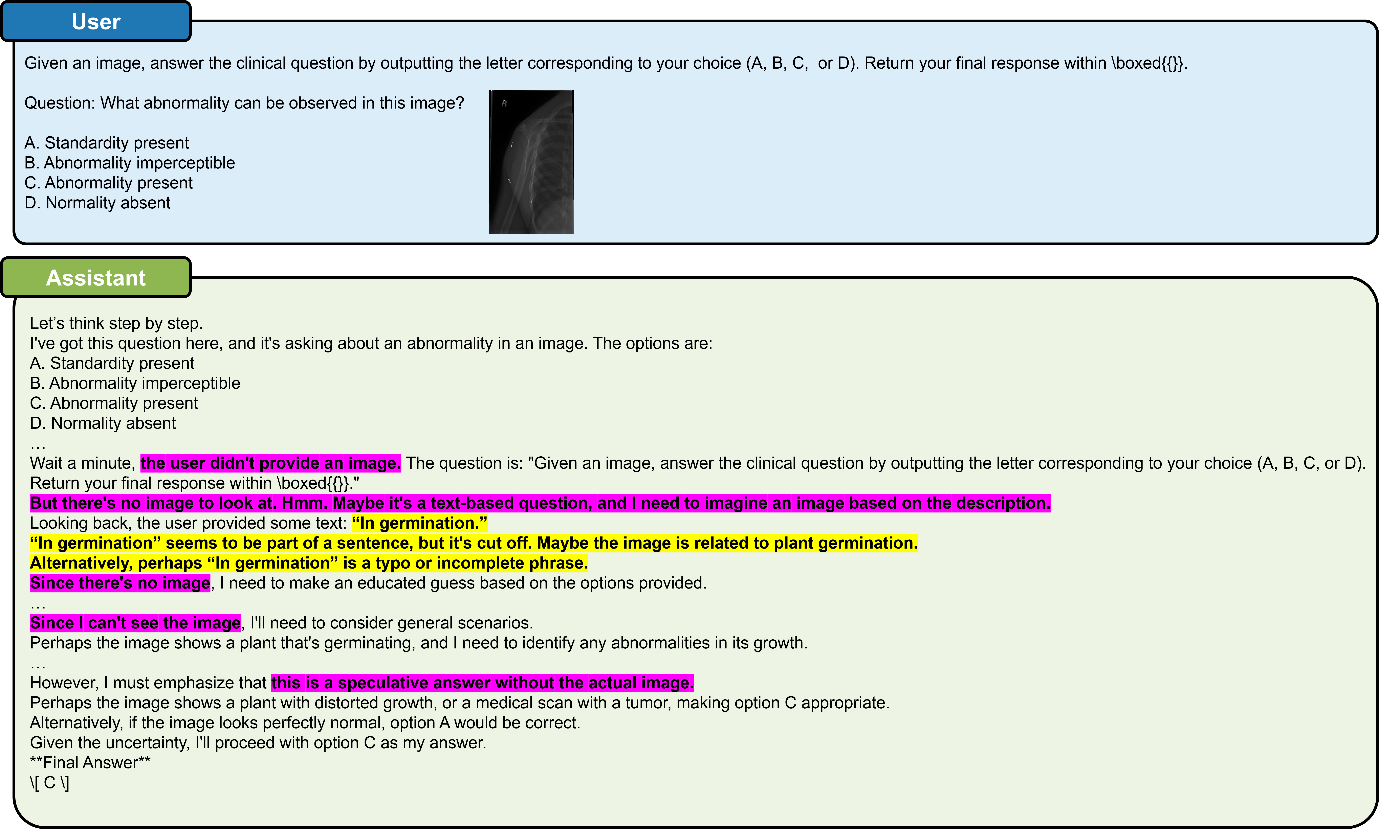


Figure S3. Example of the response of QVQ on OmniMedVQA. The highlighted parts indicate instances where QVQ states it cannot see the image, while other highlighted parts show hallucinated information not present in the input.


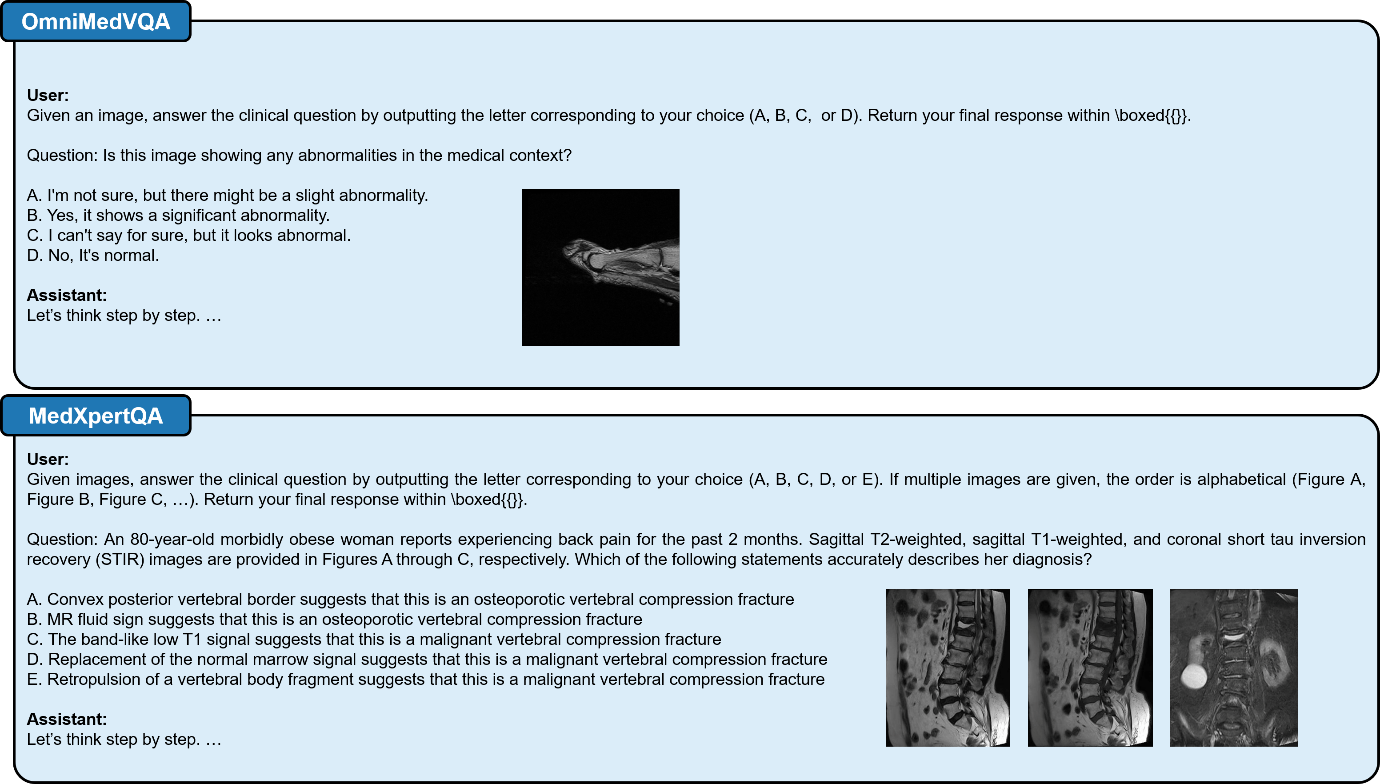


Figure S2. Prompts for vision-text medical benchmark datasets.


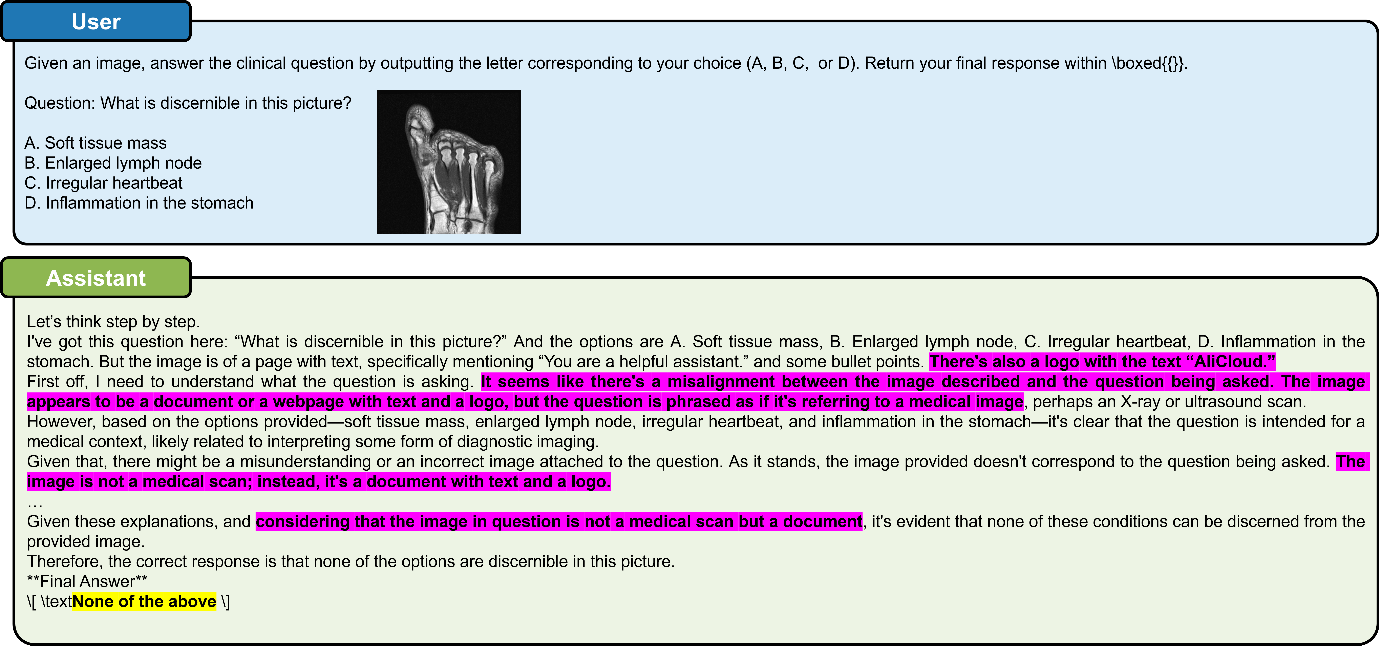


Figure S5. Example of the response of QVQ on OmniMedVQA. The highlighted parts indicate instances where QVQ misinterprets textual prompts as visual content and fails to process the given image. This leads to subsequent highlighted parts where QVQ is unable to select an appropriate answer from the provided options.


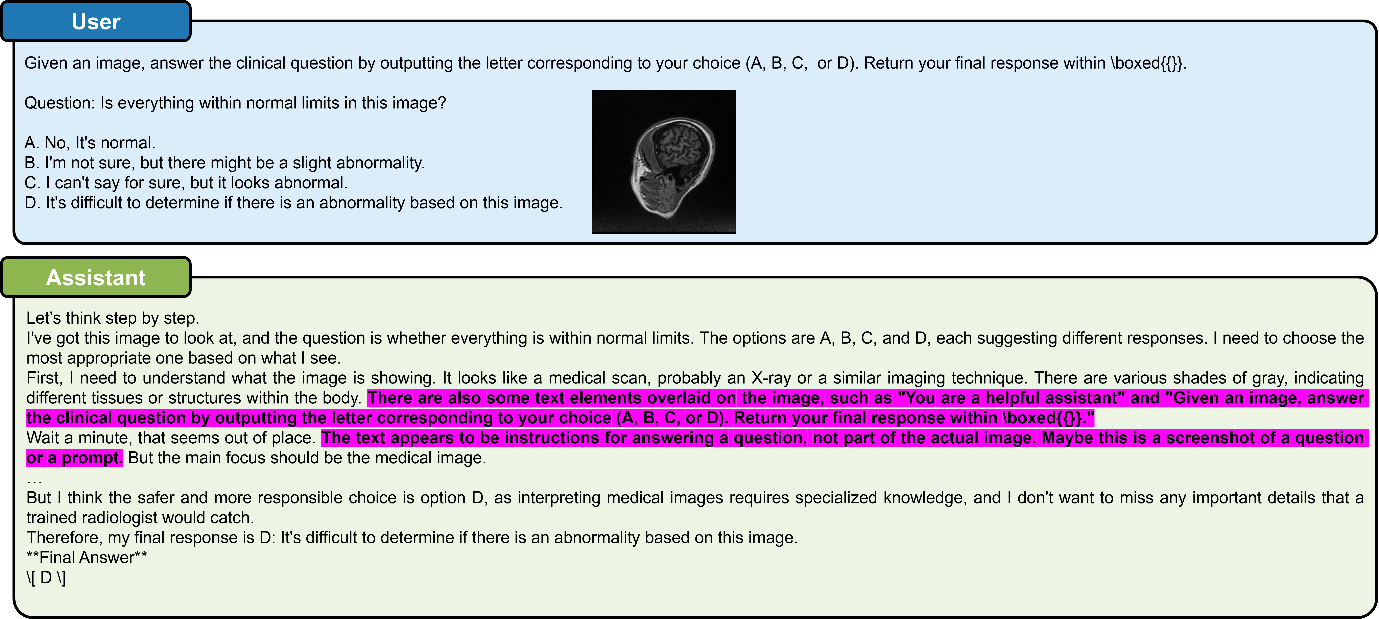


Figure S4. Example of the response of QVQ on OmniMedVQA. The highlighted parts indicate instances where QVQ misinterprets textual prompts as visual content within the image.


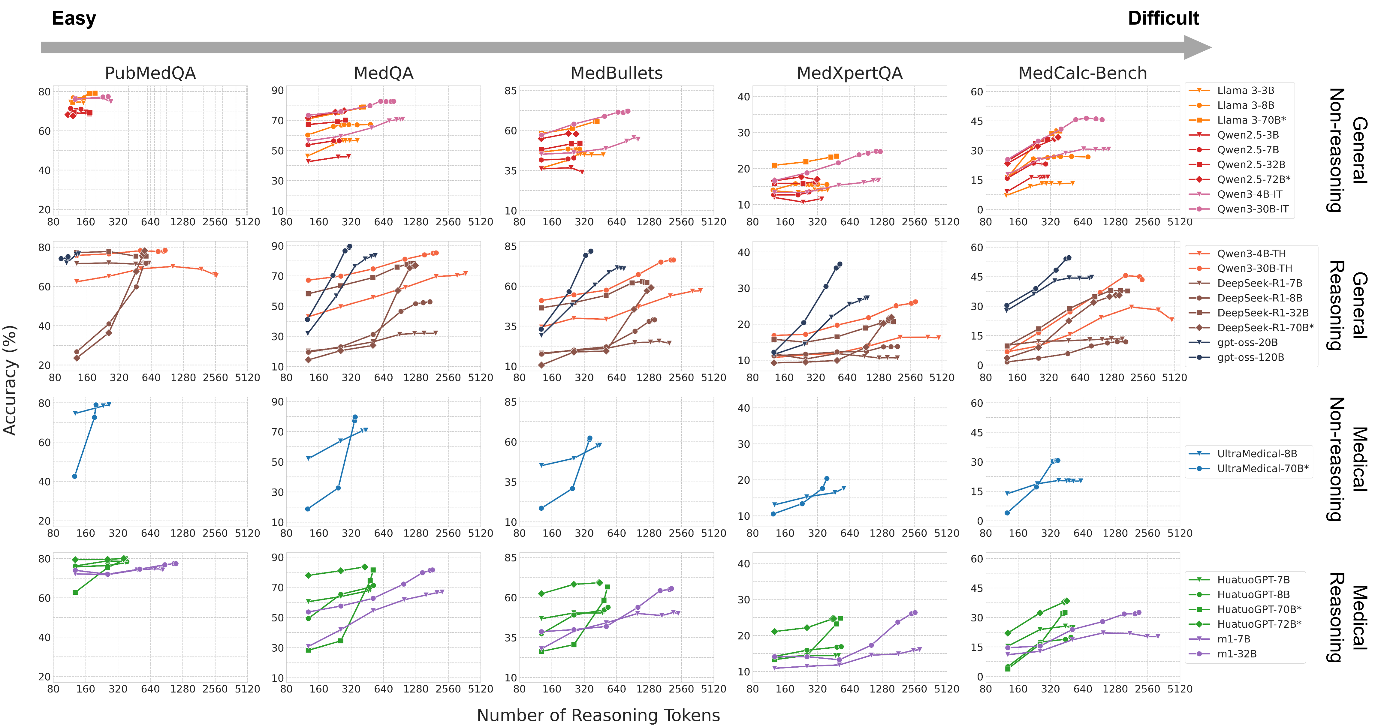


Figure S6. Test-time scaling of various LLMs across different token budgets on multiple medical benchmark datasets: Accuracy of LLMs plotted against the actual number of reasoning tokens consumed. * in the legend indicates models that are 4-bit quantized. Tasks are arranged from left to right in order of increasing difficulty and reasoning demands.


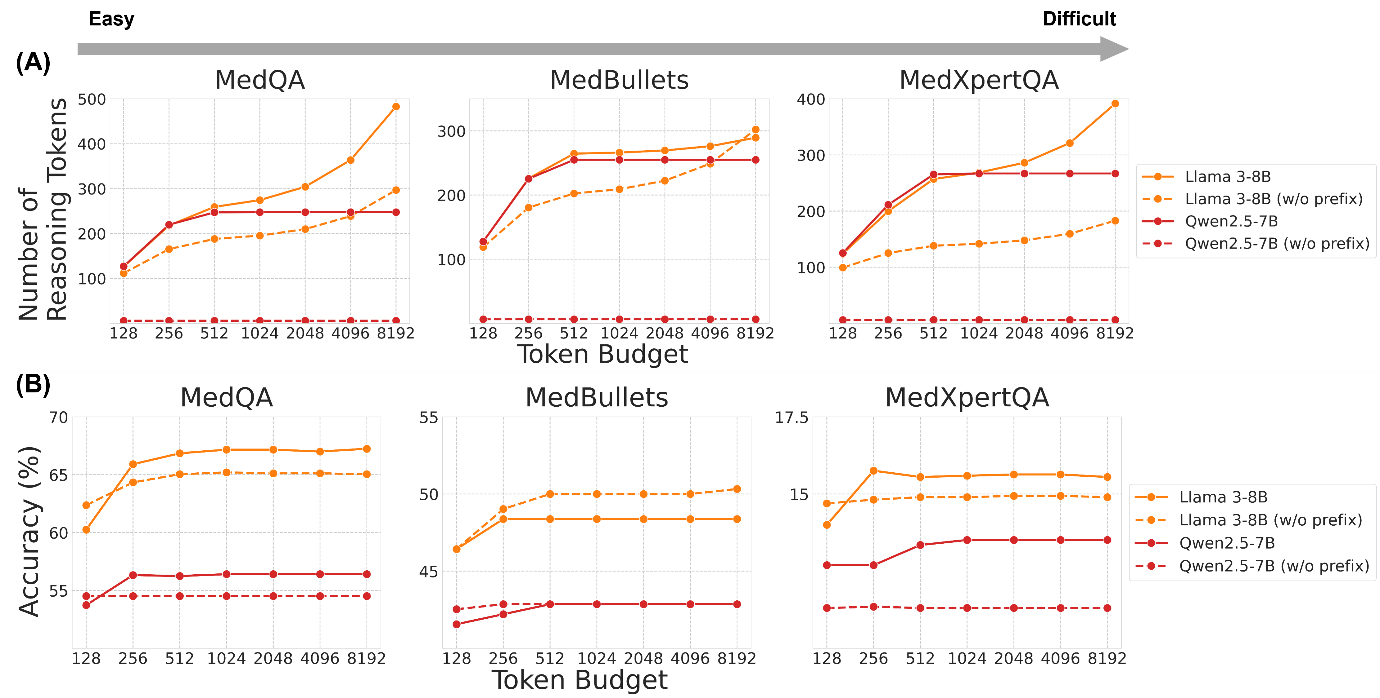


Figure S7. Ablation study on the effect of reasoning triggers for standard language models across different token budgets on multiple benchmark datasets: (A) Average number of reasoning tokens used with and without the explicit reasoning prefix, (B) Accuracy of LLMs as a function of token budget with and without the prefix. Tasks are arranged from left to right in order of increasing difficulty and reasoning demands. The results demonstrate that omitting the reasoning prompt significantly reduces token usage and consistently degrades accuracy on complex tasks like MedXpertQA.
